# Understanding *first-* and *second-order understanding*: an applied system-theoretical analysis of a police encounter with a person in crisis

**DOI:** 10.3389/fpsyg.2025.1585243

**Published:** 2026-03-17

**Authors:** Swen Koerner, Mario S. Staller, Benni Zaiser

**Affiliations:** 1 Department of Training Pedagogy and Martial Research, German Sport University Cologne, Cologne, Germany; 2 Department of Police, University of Applied Sciences for Police and Public Administration in North Rhine-Westphalia, Gelsenkirchen, Germany; 3 Independent Researcher, Aurora, ON, Canada

**Keywords:** police interaction, person in crisis, systems theory, understanding, Constraints-Led Approach, crisis intervention, de-escalation, PiC

## Abstract

Police interactions with persons experiencing mental health crisis (PiC) carry the potential for escalation. In this brief research report, we present a case study of such an encounter, examining videographic data of a widely circulated clip. The analysis follows a system-theoretical understanding of communication as a sequence of connective operations and uses the concept of turning points to identify structurally significant shifts in the interaction. The results indicate that escalation in the present example is primarily a result of communicative connection problems due to situationally divergent frames of reference and dynamic spatial compression. To stimulate practice-oriented takeaways from this analysis, we draw on the Constraints-Led Approach (CLA) as a conceptual lens for considering how these interaction problems might inform representative training design. The report thus provides both scientific insights into the structure of police interactions with PiC as well as practice-relevant impulses for the design of case-based police education and training.

## 1 Introduction

Encounters with persons experiencing mental health crisis (PiC) are challenging situations in everyday police work. Correspondingly, a continuously growing body of literature (both in Germany as well as internationally) testifies to the elevated risk of verbal and physical escalation that these encounters carry (Watson and Fulambarker, 2012; Lorey and Fegert, 2021; Wittmann et al., 2021).

While organizational and procedural aspects of such incidents have been increasingly studied (cp. Watson and El-Sabawi, 2023), the dynamics and complexities that operate within the sequential nature of the interaction remain under-researched. As a result, current training and education are limited by the corresponding gap in effectively aligning learning environments with the relational nature, within which encounters between police and PiCs play out.

The present study addresses this gap and presents a video-based interaction analysis of a police encounter that escalates and ends with the use of potentially lethal force against a PiC.

Grounded in the paradigm of case-based work (cp., Edwards, 1998), single case studies are didactically condensed depictions that illuminate typical problem constellations and recurring structural tensions, which offer meaningful indications for training design (Thistlethwaite et al., 2012). As a result, we briefly introduce the Constraints-Led Approach (CLA, Koerner et al., 2023) to translate the case study results into scenario- and case-based learning designs, which have been found to be core components of training and education in law enforcement (Buhrig, 2024), particularly in encounters involving PiC (Lavoie et al., 2022).

## 2 Methods

### 2.1 Theoretical framework

Research on police encounters with PiC shows that they often process information differently than people in not experiencing crisis. Consequently, they may respond with delays, fragmentarily and incoherently, or not at all to police-initiated conversation or commands (Morabito et al., 2012; Wittmann et al., 2021). This does not mean that PiC are deliberately ignoring or resisting police interventions. Rather, they structure the situation according to perceptual and orientational patterns that differ from those of the officers. Even clearly formulated and repeated commands such as “Put the knife down” may be, while clearly audible, failing as communicative connections, because they do not resonate with the PiC’s situational logic.

Police officers, by contrast, align the way they act with the requirements of the safety and security of everyone involved, of maintaining order as well as of law enforcement. Their training centers on assessing risk, maintaining control, enforcing necessary measures, and the use of force. During encounters with PiC, these professional motivations often clash with those of the PiC (Staller et al., 2025). While police make sense of the situation in terms of danger, controllability, duty to act, necessity as well as reasonability of the use of force, the PiC may be only motivated to reduce sensory overload or increase interpersonal distance to re-establish their comfort zone. If this discrepancy in motivation is not recognized, problems of coordination emerge that may be interpreted by officers as resistance or lack of insight. In many cases, however, these are not acts of defiance but communicative connection problems arising from the PiC’s acute, situational mode of processing reality.

System-theoretical communication theory offers an appropriate analytical framework for this study. From this perspective, communication is not understood as the mere transmission of information but as a process of establishing connection: an utterance is considered understood when another utterance or action follows that visibly connects to it (Luhmann, 1995). Such communication is not limited to verbal expression. Non-verbal communication and body language can likewise constitute communicative contributions by marking a message with informational relevance (Körner and Staller, 2023). In this view, any action that connects to a previous observation and in turn enables or prevents further connections is considered communication.

By differentiating meaning within distinctions, communication always operates through designations. Every utterance or bodily action marks a difference: for example, stepping forward or backward along the distinction proximity/distance, raising hands along threat/non-threat, changing positioning along control/lack of control, pointing to an object along relevant/irrelevant, or using a force option along intervention/non-intervention.

Approaching communication from this perspective makes it possible to distinguish between two forms of understanding, which hare also central to police crisis interactions. *First-order understanding* answers a designation simply with another designation, without acknowledging, considering, and reconstructing the underlying distinction used by the other party. The connecting operation, therefore, connects only to the (informational) surface of the utterance: For instance, a PiC might say “I hear voices” and a police officer might answer: “No, there are no voices.” The officer responds to the named element (i.e., hearing voices/not hearing voices) but remains within their own frame of reference, which guides the distinctive differentiation of meaning (i.e., real/not real). This lack of understanding does not allow the distinction introduced by the PiC (i.e., hearing voices/not hearing voices) to be the reference point of further communication. This, in turn, hinders effective de-escalation and a harm-reduced/peaceful resolution of the encounter (Birch, 2024; Krameddine and Silverstone, 2015).

In contrast, *second-order understanding*, also called *observing-understanding*, does not primarily focus on the designation at the surface level but on the distinction that the utterance marks. The guiding question is not: *Is the content true?* but rather: *What difference does the PiC make visible with this utterance?*

If, for example, a PiC says “I hear voices,” an observing-understanding response might be: “I understand that you are hearing voices speaking to you right now.” This answer relates to the PiC’s guiding distinction (hearing voices/not hearing voices) and establishes connection along that distinction, rather than along a police distinction such as real/not real. This creates a minimal but viable shared orientation without confirming the PiC’s perception to be objectively real.

System-theoretically, *observing-understanding* means observing the other person’s observation, which allows to infer within which distinction the other structures the situation. This way, the police remain capable of acting without having to adopt the PiC’s subjective reality. This is central for crisis communication, because PiC often do not communicate along police distinctions, which prevents them from processing commands or rational corrections in a way that allows connection.

Especially during interactions with PiC, the difference between *first-order* and *second-order* or *observing-*understanding is crucial, because police commands (e.g., danger/safety) often do not connect to the PiC’s situational orientation (e.g., proximity/distance). Officers then may often interpret the resulting lack of connection and subsequent failure of such rapport-building as incompliance, resistance, or lack of insight, although, system-theoretically, it concerns incompatible distinction orders.

This system-theoretical concept of communication and understanding provides a theoretical frame that explains police interactions with PiC not in terms of internal psychological states but as observable connections. Communication is understood as a sequence of verbally and non-verbally conveyed designations that each mark a distinction, indicating how the participants structure the situation.

The following section presents a case-study that illustrates how problems of connection arise and escalate during real-world interactions between PiC and police.

### 2.2 Case description

The analysis is based on a publicly accessible video clip that shows a police encounter with a PiC. We selected the case because public reports indicate that the person involved experiencing a mental health crisis.

The excerpt shows a sequence of approximately 77.2 s, in which four police officers confront a PiC holding a knife. The police have already arrived on scene. Initially, they keep a distance of several meters. The clip ends with two gunshots, after which the PiC falls to the ground. The camera captures the scene from an elevated perspective, likely from an upper-floor window. Both PiC and the involved officers, as well as parts of their movements and formation changes, can be seen. The events leading up to the recording, other contextual information, and parallel communication, such as radio traffic, are not captured. The analysis therefore refers solely to the situationally observable actions documented in the video.

### 2.3 Ethical considerations

The scientific analysis of real video recordings of violent or crisis situations raises fundamental ethical questions (Nassauer and Legewie, 2018). In the present study, this tension is addressed by deliberately refraining from moral, legal, or procedural evaluation. The goal is not to identify individual misconduct or assign blame but to reconstruct structural interaction patterns that contributed to the escalation.

The analysis focuses strictly on observable communication and behaviors, without psychological attributions or speculations about internal states. From an ethical perspective, we conclude that the analysis is justified because of its value for understanding and improving interactions between police and PiC.

### 2.4 Analytical procedure

The analysis follows a video-based, sequential approach as established in research on violence and interaction (Nassauer and Legewie, 2018; Nassauer, 2022). The video clip was fully transcribed and time-coded. The transcription includes verbal utterances as well as observable behaviors (e.g., spatial positioning, movement directions, gestures) of both the officers and the PiC. The aim was to reconstruct the course of interaction along the temporal logic of the documented events. For the analysis, the PiC is designated as C (citizen), while the involved police officers are referred to as P1, P2, etc. Time stamps in brackets mark the corresponding moments in the video.

Based on the the system-theoretical understanding of communication as a process of establishing connection (see above, see section “2.1 Theoretical framework”), verbal utterances and observable behaviors of all parties are treated as equal communicative contributions, as long as they connect to prior designations and mark a distinction.

The concept of turning points is at the core of the analysis. Each turning point represents a key moment during the encounter, which triggers a shift in the dynamics of subsequent action. These moments signify visible changes in participants’ behaviors and can orient the interaction toward physical violence or away from it: “Turning points mark a noticeable change in the type of actions; they introduce a segment of actions which orient the interaction more markedly toward physical violence or away from it (Weenink et al., 2022, p. 582).”

The analysis follows two steps: (1) a descriptive reconstruction of the interaction sequences surrounding the turning points (with time stamps in seconds), and (2) a system-theoretical interpretation with regard to the distinctions marked and the form of understanding (*first*- or *second-order*). The coding of the full sequence was initially conducted by the first author, using an inductive, material-driven approach (Braun and Clarke, 2021). Following the first cycle of coding, the full set of codes was discussed by the first and second author to ensure intercoder agreement. The systems-theoretical interpretation of the data was drafted by the first author and subsequently discussed and consented between all authors.

## 3 Results

### 3.1 Turning pint 1: “What do you have there?”

#### 3.1.1 Descriptive interaction sequence

At the beginning of the video, C is holding an unidentified object in both hands. P1 addresses C directly and says, “Stay calm” (00:01.5). This is followed by two commands: “Put it down immediately” (00:02.9) and, repeated louder, “put it down immediately” (00:04.3–00:05.4). Immediately afterwards, P2 asks: “What do you have there?” (00:05.5).

In response, C pulls a knife out of a sheath (00:06.9–00:09.5). C now holds the knife in the left hand and the empty sheath in the right (00:09.5). Immediately thereafter, P1, P2, and P3 shout almost simultaneously (00:10.4–00:11.3). P1 begins with “put the knife…,” P2 shouts “knife!”, P3 produces a loud but unintelligible shout, and P1 finishes the utterance with “…away!” (00:11.3). At the same time, P1 points with an outstretched arm toward the knife (00:10.7), while P2 draws the firearm and points it at C (00:10.9). C then slightly raises the right hand with the sheath (00:11.6).

Subsequently, the interaction continues for about 40 s without major observable changes: the officers repeatedly direct C to put the knife down, while C repeatedly murmurs “doesn’t matter” (00:40.0–00:41.9) and “nothing changes” (00:44.7–00:45.5).

#### 3.1.2 System-theoretical interpretation

Already during the first few seconds, several central distinctions become visible that shape the subsequent interaction. The utterances by P1 (“Put it down”) constitute designations within the danger/safety distinction that guides the officers. P2’s question (“What do you have there?”) additionally marks a distinction between uncertainty/clarification. It signals that the situation is initially contingent and not yet clearly assessable.

C’s reaction by drawing the knife is a behavioral designation that connects to the previous police commands but transforms their meaning: C identifies the object without explaining why it is being held or what it actually means to C.

The simultaneous shouts (“Knife!”, “Put the knife away!”) are reactions to a *first-order* observation: the officers align their communication and actions with the named object (“knife”) but fail to consider C’s perspective, remaining within the logic of the danger/safety distinction. The non-verbal behaviors of pointing and drawing a weapon are communicative designations that reinforce that distinction.

*Second-order understanding* in the sense of reconstructing C’s distinction is not yet discernible. C does not formulate their own explicit framing distinction. Instead, they act in a way that cannot be clearly integrated into Ps’ danger/safety distinction. The officers, therefore, address only their own orientation. It already becomes apparent that both sides operate along different distinction orders, without creating a shared basis of connection.

### 3.2 Turning point 2: quarter circle

#### 3.2.1 Descriptive interaction sequence

Approximately 50 s into the clip, the course of the interaction changes significantly. C clearly tells the officers: “Go away from here” (00:49.9–00:51.0). While saying this, C rolls up the sleeves (00:50.5) and places the knife against their right forearm (00:55.7). C repeats the demand for distance: “Go away from here” (00:55.5–00:56.4).

At the same time, C moves toward P1, extending their right arm, keeping their left arm in a lowered position (00:56.4–01:00.4). Between 00:56.5 and 01:06.2, a delivery van reverses out of a parking space and creates a visual distraction.

P1 continues to direct C: “Put the knife down” (00:56.3–00:57.4), partially overlapping with C’s distance demands. P2 shouts “knife down” (00:57.4–00:57.8), re-aims the firearm, and moves toward P1, crossing paths with P3. C repeats “go away” (00:58.5–00:59.0) and “go away, out of here” (01:00.0–01:01.2). Afterwards, C produces unintelligible sounds (01:03.8–01:05.0) and begins to bounce in place (01:05.5–01:07.1).

During this time, P1, P2, P3, and P4 reposition themselves and form a quarter circle around C between 01:04.1 and 01:08.6.

#### 3.2.2 System-theoretical interpretation

With C’s first explicit utterance “go away from here,” a new distinction becomes visible: proximity/distance. This distinction remains dominant in the subsequent sequence. Rolling up the sleeves and placing the knife against the forearm are behavioral designations that can be interpreted as self-focusing or attempts to regulate overload. In terms of system-theory, they also manifest needs for distance and control, but not along the logic of the danger/safety distinction that the police officers are following.

The commands “put the knife down” reproduce the danger/safety distinction and do not address the distance logic that C is operating under. Forming a quarter circle as they approach C, a purely non-verbal communication operation, also marks a police distinction of control/uncontrollability. The officers attempt to structure the spatial dynamics. From C’s perspective, however, this may produce a sense of entrapment that contradicts their expressed demand for distance.

This turning point is exclusively characterized by the officers’ *first-order understanding*: they respond only to the presence of the “knife,” not to the utterance “go away.” C, in turn, does not show readiness to connect to the police commands. Both sides continue their respective distinction orders in parallel. The absence of *second-order* or *observing-understanding* means that no shared focus for the interaction is established. The interaction continues to escalate.

### 3.3 Turning point 3: “pepper!”

#### 3.3.1 Descriptive interaction sequence

At the beginning of this segment, C once again says: “Go away from here” (01:06.9–01:07.6). P2 responds with an overlapping “Yeah” (01:07.1–01:07.5). C then produces unintelligible sounds (01:07.7–01:08.8). P2 then calls out “pepper!” (01:08.6–01:09.2), upon which P4 immediately deploys pepper spray (01:09.2–01:10.5).

C reflexively raises the right hand, turns away (01:10.5), then turns back toward the officers and runs directly at P4 (01:11.1–01:13.5). P4 moves backward, draws the firearm, and repeatedly shouts: “Drop the knife, drop the knife…” (01:11.5–01:13.3). At 01:13.3–01:14.3, P4 fires two shots. C collapses.

#### 3.3.2 System-theoretical interpretation

During this turning point, hardly any verbal connection can be heard. C continues to repeat their demand for distance, while the police shift their operative distinction toward intervention/enforcement of measures. The exclamation “pepper” announces the transition to the next level of force. Simultaneously, it communicates implicitly that the situation has qualitatively changed.

The use of pepper spray is a non-verbal communication following the logic of the officers’ understanding of C’s actions based on their *first-order observation* of an intervention/enforcement of measures distinction. C cannot respond verbally anymore. Raising an arm defensively, turning away, and running toward P4 are then reactions, which C reinforces the intervention/enforcement and activates the danger/safety distinction. The officers continue to issue commands (“drop the knife”). At the same time, C continues to not process these directions. The possibility to achieve a *second-order understanding* is eliminated.

Ultimately, the use of firearms illustrates the complete breakdown of mutual connection and understanding: both sides now act exclusively along their respective behavioral and operational logics. Communication ceases, what remains is physical violence.

## 4 Discussion

### 4.1 Summary of findings

The analysis shows escalation as the result of a gradual accumulation of small, often barely perceptible breakdowns of understanding. These breakdowns occur where the police and the PiC operate incompatibly along different distinction logics: danger/safety on the side of the police versus proximity/distance and overload/relief on the PiC’s side. The system-theoretical perspective makes it possible to describe these dynamics as interactional phenomena without individualizing blame. The central questions are: Which designations are made? Which distinctions do they mark? And does *first-order* or *second-order* observation and understanding occur? The turning points reconstructed in the case study mark those moments in which structural decoupling becomes visible, and the interaction moves into a space where de-escalatory connection becomes increasingly unlikely.

### 4.2 Limitations

The study has several limitations. First, it is based on a single video-documented incident in a specific national context. The findings are therefore not generalizable. From a case-based perspective (Buhrig, 2024), however, the aim is not to produce frequency statements, but to reconstruct exemplary interaction logics that can be made productive for education and training. Second, the chosen approach does not provide direct access to the subjective experiences of those involved. In line with the system-theoretical framework, no mental states are reconstructed. We analyzed only observable designations, distinctions, and connection operations. Third, videographic data can capture spatial and acoustic complexity only in fragments (Weenink et al., 2022). Relevant stimuli outside the frame or off mic remain invisible. Fourth, as audio- and video-based analyses are inherently constrained by the fixed point of view of the recording, blind spots and potential interpretation bias should be taken into account (Boivin et al., 2023).

### 4.3 Outlook

Despite these limitations, the present case study illustrates how escalation in encounters between police and PiC can emerge from a gradual accumulation of connection losses, driven by divergent distinction logics and dynamically changing spatial conditions. For police education, in particular, this raises a practical question (Krameddine and Silverstone, 2015): How can training be designed so that officers can work with these interactional challenges in a realistic but safe way?

The analysis suggests that it may be useful to focus less on presumed inner states and more on the situational conditions under which particular patterns of understanding and non-understanding become likely. This is consistent with approaches from ecological dynamics (Koerner et al., 2023), in which behavior is understood as emerging from the interplay of constraints rather than from isolated individual traits. One promising framework in this tradition is the Constraints-Led Approach (CLA), which conceptualizes human behavior as shaped by the interaction of organismic, environmental, and task constraints (Renshaw and Chow, 2019).

From this perspective, the present case can be read as a configuration of constraints: high organismic load on both the PiC and the officers, environmental factors such as spatial compression and multiple simultaneous speakers, and task demands related to danger prevention and enforcement. In training, such constellations can be modeled by designing scenarios in which actors in the role of PiC consistently maintain their own frame of reference (e.g., proximity/distance) and do not reliably respond to commands. Debriefings can then focus on how specific police actions – approach, encirclement, parallel speech, or ignoring distance demands – increased or reduced load and opened or closed possibilities for *second-order* observing/understanding.

In this way, insights from the present system-theoretical analysis can be linked to a constraints-oriented training logic. The CLA does not offer a ready-made solution, but it provides a structured way to design and vary scenarios (Koerner et al., 2023) so that officers can physically and communicatively experience the tension between load and de-load, and practice maintaining communicative connection with PiC under operational conditions. Future work should develop and empirically evaluate such CLA-informed training formats, building explicitly on case material of the kind analyzed here.
